# The mortality for the implantable cardiac defibrillator in nonischemic cardiomyopathy: An updated systematic review and meta‐analysis

**DOI:** 10.1002/clc.23907

**Published:** 2022-09-03

**Authors:** Wenfeng He, Cheng Xue, Jiankang Zheng, Zhuang Shuai

**Affiliations:** ^1^ Cardiovascular Disease Laboratory, Department of Cardiology Affiliated Hospital of North Sichuan Medical College Sichuan Province China

**Keywords:** all‐cause mortality, cardiovascular mortality, implantable cardiac defibrillator, meta‐analysis, nonischemic cardiomyopathy, sudden cardiac death

## Abstract

The implantable cardiac defibrillator (ICD) is common for the management of nonischemic cardiomyopathy (NICM). Mortality is a crucial issue for patients with NICM. We can understand the mortality events of ICD versus medicine treatment via a systemic review and meta‐analysis of randomized clinical trials. The comparison between ICD treatment and medicine treatment was performed to find if the ICD treatment can be associated with lower relative risk and hazard ratio of mortality than the medicine treatment. In addition, the different kinds of mortality events were analyzed for the ICD treatment. After a restricted selection, 9 studies with a total of 4001 NICM patients were enrolled. The focused outcome was the events of all‐cause mortality, sudden cardiac death, and cardiovascular death. The results showed that ICD treatment might be associated with lower relative risk and hazard ratio of all‐cause mortality and sudden cardiac death. However, the relative risk and hazard ratio of cardiovascular mortality was not significantly different between ICD treatment and medicine treatment. In the current meta‐analysis, the ICD treatment might show a lower relative risk and hazard ratio of all‐cause mortality and sudden cardiac death when compared with medicine treatment. However, no significant differences were observed in cardiovascular mortality between ICD and medicine treatment.

## 1 INTRODUCTION

The implantable cardiac defibrillator (ICD) is an important treatment to relieve the heart failure symptoms of systolic dysfunction and ischemic cardiomyopathy.^1^ The ICD is recommended for ischemic cardiomyopathy with reduced ejection fraction and systolic dysfunction symptoms. For the nonischemic cardiomyopathy (NICM), the past largest randomized DANISH study^2^ failed to find the benefit of higher overall survival for the NICM. Therefore in 2016–2018, several meta‐analytic studies^3^, ^4^, ^5^, ^6^, ^7^, ^8^, ^9^, ^10^, ^11^, ^12^ of randomized clinical trials tried to find evidence to support the advantages of ICD to reduce all‐cause mortality and sudden cardiac death, which were the major causes of deaths of NICM.^9^

Most of the meta‐analysis studies showed that the ICD treatment might be associated with lower relative risk (risk ratio) (RR) and log‐transformed hazard ratio (log HR) for all‐cause mortality. However, the evidence was limited by the conflicting results between the positive results of the Sudden Cardiac Death in Heart Failure Trial (SCD‐HeFT)^1^ and Comparison of Medical Therapy, Pacing, and Defibrillation in Heart Failure (COMPANION) trial^13^ and the negative results from other trials. The trials of negative results exclusively enrolled patients with NICM. The positive trials (SCD‐HeFT trial and COMPANION) included patients with ischemic cardiomyopathy and NICM. SCD‐HeFT and COMPANION trails studied the effect of NICM in the sub‐analysis. Therefore, the results might be biased by the mixture characteristics of trials. In addition, the benefits of the ICD for sudden cardiac death and cardiovascular mortality might not be so consistent. Therefore, the primary prevention of sudden cardiac death and the reduction of mortality still needs more updated studies to be enrolled in the meta‐analysis, which can clarify the role of ICD for reducing the mortality, especially for the not conclusive roles in cardiovascular mortality and sudden cardiac death.

In the current meta‐analysis, we would enroll more updated randomized clinical trials for the ICD versus medicine treatment (optimal medicine treatment) in the patients of NICM, especially after 2017. According to previous meta‐analysis results,^3^, ^4^, ^5^, ^6^, ^7^, ^8^, ^9^, ^10^, ^11^, ^12^, ^14^ we hypothesized that the ICD treatment might show significantly superior effects to reduce all‐cause mortality, sudden cardiac death, and cardiovascular death when compared with medicine treatment. The RR and log HR would be the index to evaluate the primary prevention of mortality for the ICD treatment.

## 2 METHODS

### 2.1 Literature search and selection criteria

The following keywords of “defibrillator,” “ICD,” “nonischemic,” “cardiomyopathy,” “trials,” “randomized,” “clinical,” “controlled,” “treatment,” “therapy,” “efficacy” or “outcome,” “comparison,” “versus,” “implantable,” and “cardiac” were used to search and collect the related prospective RCT articles in the PubMed, ScienceDirect, EmBase, Web of Science, and the Cochrane Central Register of Controlled Trials (CENTRAL). The articles were limited to those published or e‐published online before May 2022.

The eligible criteria of enrolled studies were as follows: (1) The comparisons between ICD therapy and medicine (pharmacological) alone treatment for the NICM patients. (2) The studies investigating the baseline and posttreatment outcome profile for the mortality, including all‐cause, cardiovascular mortality and sudden cardiac death. (3) The studies with detailed data of outcome in the perspective of mortality. (4) Restriction to be published in the English language style in the journals of science citation index database. (5) Randomized clinical trials. (6) The trials of the patients without a family history of a fatal tachyarrythmias or cardiac arrest.

### 2.2 Quality assessment and data extraction

The systematic review and meta‐analysis study was conducted based on the Cochrane Handbook for Systematic Reviews and Interventions. The results were reported according to the preferred reporting items for systematic reviews and meta‐analyses (PRISMA) guidelines.^15^ The risk of bias for each study was assessed by the bias arising from the randomization process, bias due to deviations from intended interventions, bias due to missing outcome data, bias in the measurement of the outcome, and bias in the selection of the reported result. We extracted the following data from the eligible articles. First, the all‐cause mortality event and patient number after ICD therapy and medicine treatment for NICM. Second, the sudden cardiac death event and patient number after ICD therapy and medicine treatment for NICM. Third, the cardiovascular mortality event and patient number after ICD therapy and medicine treatment for NICM.

### 2.3 Data extraction and critical appraisal

Two reviewers reviewed the abstracts to screen the articles and then independently evaluate the full‐text version of the selected citations. Then the two reviewers performed the extraction of clinical outcome data from text, tables, and figures of the enrolled articles independently. The enrolled articles basically had the data of all‐cause mortality, sudden cardiac death, and cardiovascular mortality in the full‐text content. The risk of bias was evaluated by the bias arising from the randomization process, bias due to deviations from intended interventions, bias due to missing outcome data, bias in the measurement of the outcome, and bias in the selection of the reported result. Then all authors performed collaborative reviews to resolve any discrepancies to achieve the strong agreement (κ = .9). All authors participated to review the final results.

### 2.4 Meta‐analysis and statistical analysis

For all‐cause mortality, cardiovascular mortality, and sudden cardiac death, we generated pooled estimates of relative risks (RR) or hazard ratios (HR) along with the associated 95% confidence interval (CI). Due to the lack of patient‐level data, we used summary statistics for each trial by extracting the reported HRs. In studies without HRs and respective 95% confidence intervals, the Kaplan–Meier survival curves were calculated for each individual study and used the Guyton algorithm to reconstruct individual patient data for subsequently obtaining an estimate of HRs and 95% CI in survival analysis. We used the Cochrane Collaboration Review Manager Software Package (Rev Man Version 5.4) to perform the meta‐analyses. We reported Mantel–Haenszel RR using DerSimonian and Laird's random‐effect models by using the summary statistics. The data was transformed to the log‐HRs using the HR and the start of 95% CIs in the Rev Man calculation function. The risk estimates of individual studies were combined via the inverse variance weighted averages of log HRs in the random‐effects model. In addition, the random and fixed effects models were used with inverse variance function weighted log HR. The ICD therapy and medicine treatment were compared with each other to find if ICD therapy will be superior in decreasing the events of all‐cause mortality, sudden cardiac death, and cardiovascular mortality. The *χ* ^2^ tests were used to study heterogeneity between enrolled studies. The derived *I* ^2^ statistic was used to estimate statistical heterogeneity of studies included in the meta‐analysis. The cutoff value of the Higgins *I* ^2^ index was as follows: 0%–40%: might not be important; 30%–60%: may represent moderate heterogeneity*; 50%–90%: may represent substantial heterogeneity*; 75%–100%: considerable heterogeneity.^16^ Based on Cochrane Handbook section 10–10–4–1, it clearly states “The choice between a fixed‐effect and a random‐effects meta‐analysis should never be made on the basis of a statistical test for heterogeneity.” 0% of I‐statistics only implies there is no statistical heterogeneity but it is still inevitable to have methodologic/conceptual heterogeneity unless authors can justify the consistency of methodologic/conceptual heterogeneity across included trials. Otherwise, it would be more appropriate to use the random‐effects model instead of the fixed‐effects model. Therefore, we used the random effects model in the current meta‐analysis. All *p* values were two‐sided. Publication bias was assessed using a funnel plot and Eggers's test to find if there was a symmetric distribution of pooled studies.

## 3 RESULTS

### 3.1 Description of studies

**Figure 1:**
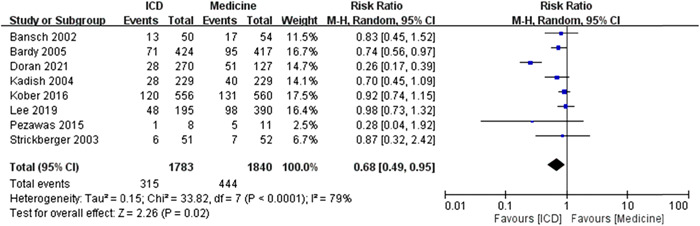
The forest plot of risk ratio [RR] for the meta‐analysis results of all‐cause mortality [ICD vs. medicine treatment]. The ICD treatment showed significantly lower RR to have the events of all‐cause mortality when compared to the medicine treatment (statistically significant). A significant heterogeneity was noted

A total of 2817 articles from database search and 59 additional records from other sources were initially collected. Within these articles, 721 duplicate articles were removed. Then the relevance of abstracts and titles were screened for the residual 2155 articles. The 2113 articles were excluded after screening the relevance. Full‐text contents of screened 42 articles were assessed for the eligibility. After the assessment of full‐text content, 33 articles were discarded (Figure S1). The qualitative analysis of residual nine articles was performed and the residual nine studies were included in this meta‐analysis. The PRISMA flow diagram of current meta‐analysis was presented as Figure 1. In addition, the detailed information of the enrolled 9 studies were summarized in Table 1. The risk of bias assessment showed that three studies have some concerns of bias from the selection of reported results (Figure S2). The funnel plot of enrolled studies showed a symmetric distribution (Figure S3).

### 3.2 RR of all‐cause mortality of the ICD versus medicine treatment for NICM

The *I* ^2^ was 79%, which revealed the high heterogeneity. The test for overall effect was *Z* = 2.26 (*p* = .02) and the meta‐analysis results showed significant difference of RR of all‐cause mortality events between ICD and medicine treatment under random effects model (Figure 1).

**Table 1:** Summary of enrolled studies for the mortality of ICD treatment versus medicine treatment on NICM patients

	NICM patients (ICD vs. medicine)	Inclusion criteria and ejection fraction	Concomitant medicine	Outcome
Bansch 2002 (Multicenter, Germany)	50 (86% male; 52 ± 11 years old, 20% atrial fibrillation) vs. 54 (74% male, 52 ± 11 years old, optimal medicine treatment; 11% atrial fibrillation)	Recent‐onset Idiopathic dilated cardiomyopathy ejection fraction ≤30% NYHA II–III Left ventricle ejection fraction (24 ± 7%)	Angiotensin‐converting enzyme inhibitor/angiotensin receptor blocker (96%) beta‐blocker (4%)	5.5 ± 2.2 years follow‐up 1‐year mortality in medicine group (4%) outcome index: all‐cause mortality cardiovascular mortality
Bardy 2005 (Multicenter)	424 (77% male; 60 years old, 17% atrial fibrillation) vs. 417 (77% male, 60 years old, optimal medicine treatment+amiodarone; 14% atrial fibrillation)	Left ventricle ejection fraction ≤35% NYHA class II–III Left ventricle ejection fraction (25%)	Angiotensin‐converting enzyme inhibitor/angiotensin receptor blocker (97%) beta‐blocker (69%) mineralocorticoid receptor antagonist (20%)	3.8 years follow up 1‐year mortality in medicine group (8% in amiodarone 4% in placebo) outcome index: all‐cause mortality
Bristow 2004 (Multicenter)	270 (70% male; 67 years old) vs. 127 (70% male, 67 years old, optimal medicine treatment)	NYHA class III or IV heart failure resulting from either ischemic or nonischemic cardiomyopathy, left ventricular ejection fraction of 35% or less, an electro‐cardiographically measured QRS interval of at least 120 msec and a PR interval of more than 150 msec, sinus rhythm Left ventricle ejection fraction (20%)	Angiotensin‐converting enzyme inhibitor/angiotensin receptor blocker (90%) beta‐blocker (68%) loop diuretic (94%) spironolactone (55%)	1.3 years follow‐up outcome index: all‐cause mortality all‐cause hospitalization
Doran 2021 (Multicenter)	270 (70% male; 67 years old) vs. 127 (70% male, 67 years old, optimal medicine treatment)	NYHA Class III or IV heart failure resulting from either ischemic or nonischemic cardiomyopathy, left ventricular ejection fraction of 35%	Angiotensin‐converting enzyme inhibitor/angiotensin receptor blocker (90%) beta‐blocker (68%)	1.3 years follow‐up outcome index: all‐cause mortality cardiovascular mortality sudden cardiac death
Kadish 2004 (Multicenter, USA)	229 (73% male; 58 years old, 23% atrial fibrillation) vs. 229 (70% male, 58 years old, optimal medicine treatment; 26% atrial fibrillation)	Left ventricle ejection fraction ≤35% NYHA Class I–III non‐sustained ventricular tachycardia or frequent premature ventricle complex Left ventricle ejection fraction (21%)	Angiotensin‐converting enzyme inhibitor/angiotensin receptor blocker (97%) beta‐blocker (85%)	2.4 ± 1.2 years follow‐up 1‐year mortality in medicine group (6%) outcome index: all‐cause mortality cardiovascular mortality sudden cardiac death
Kober 2016 (Multicenter, UK)	556 (73% male; 64 years old, 24% atrial fibrillation) vs. 560 (72% male, 63 years old, optimal medicine treatment; 20% atrial fibrillation)	Left ventricle ejection fraction ≤35% NYHA Class II–IV N‐terminal pre‐brain natriuretic peptide >200 Left ventricle ejection fraction (25%)	Angiotensin‐converting enzyme inhibitor/angiotensin receptor blocker (97%) beta‐blocker (92%) mineralocorticoid receptor antagonist (58%) amiodarone (6%)	5.6 years follow up 1‐year mortality in medicine group (4%) outcome index: all‐cause mortality cardiovascular mortality sudden cardiac death
Lee 2019 (Multicenter)	195 (47% male; 58.1 years old) vs. 390 (41% male, 57 years old, optimal medicine treatment)	Left ventricle ejection fraction ≤35% NYHA Class II–IV	Angiotensin‐converting enzyme inhibitor/angiotensin receptor blocker (90%) beta‐blocker (93%) arpirin (73%)	6 years follow‐up outcome index: all‐cause mortality cardiovascular mortality sudden cardiac death
Pezawas 2015 (Single center Austria)	19 (100% male; 64 years old, 22% atrial fibrillation)	NYHA Class III–IV listed for heart transplantation no history of supraventricular tachycardia or cardiac arrest left ventricle ejection fraction (19%)	Angiotensin‐converting enzyme inhibitor/angiotensin receptor blocker (100%) beta‐blocker (82%) mineralocorticoid receptor antagonist (48%) amiodarone (24%)	0.9 year follow‐up outcome index: all‐cause mortality
Strickberger 2003 (Multicenter)	51 (67% male; 58 years old) vs. 52 (74% male, 60 years old, optimal medicine treatment)	Left ventricle ejection fraction ≤35% NYHA class I–III non‐sustained ventricular tachycardia or frequent premature ventricle complex	Angiotensin‐converting enzyme inhibitor/angiotensin receptor blocker (85%) beta‐blocker (52%) mineralocorticoid receptor antagonist (20%)	2 years follow‐up 1‐year mortality in medicine group (4%) outcome index:all‐cause mortality cardiovascular mortality sudden cardiac death

Abbreviations: ICD, implantable cardiac defibrillator; NICM, nonischemic cardiomyopathy.

### 3.3 Log HR of all‐cause mortality of the ICD versus medicine treatment for NICM

**Figure 2:**
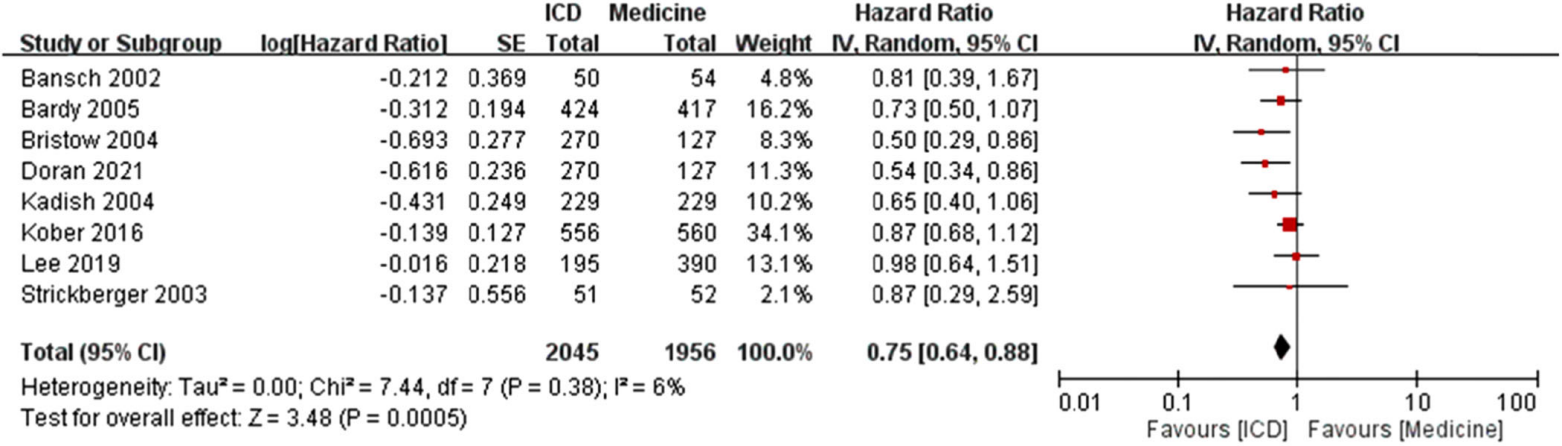
The forest plot of log HR for the meta‐analysis results of all‐cause mortality [ICD vs. medicine treatment]. The ICD treatment showed significantly lower log HR to have the events of all‐cause mortality when compared to the medicine treatment (statistically significant). CI, confidence interval; HR, hazard ratio; ICD, implantable cardiac defibrillator

The *I* ^2^ was 6%, which revealed the low heterogeneity. The test for overall effect was *Z* = 3.48 (*p* = .0005) and the meta‐analysis results showed significant difference of log HR of all‐cause mortality events between ICD and medicine treatment under random effects model (Figure 2).

### 3.4 RR of sudden cardiac death of the ICD versus medicine treatment for NICM

**Figure 3:**
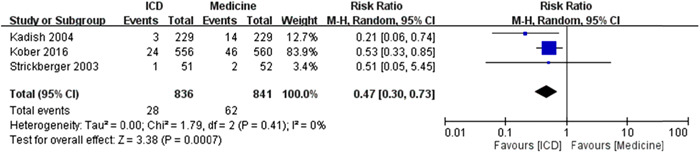
The forest plot of RR for the meta‐analysis results of sudden cardiac death [ICD vs. medicine treatment]. The ICD treatment showed significantly lower RR to have the events of sudden cardiac death when compared to the medicine treatment (statistically significant). CI, confidence interval; ICD, implantable cardiac defibrillator; RR, risk ratio

The *I* ^2^ was 0%, which revealed extremely low heterogeneity. The test for overall effect was *Z* = 3.38 (*p* = .0007) and the meta‐analysis results showed a significant difference of RR of sudden cardiac death events between ICD and medicine treatment under random effects model (Figure 3).

### 3.5 Log HR of sudden cardiac death of the ICD versus medicine treatment for NICM

**Figure 4:**
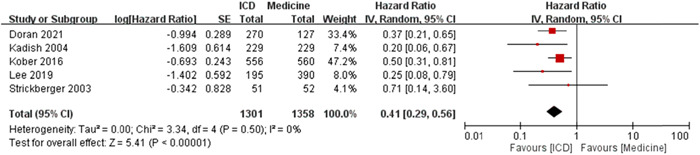
The forest plot of log HR for the meta‐analysis results of sudden cardiac death [ICD vs. medicine treatment]. The ICD treatment showed significantly lower log HR to have the events of sudden cardiac death when compared to the medicine treatment (statistically significant). CI, confidence interval; HR, hazard ratio; ICD, implantable cardiac defibrillator; SE, standard error

The *I* ^2^ was 0%, which revealed extremely low heterogeneity. The test for overall effect was *Z* = 5.41 (*p* < .00001) and the meta‐analysis results showed significant difference of log HR of sudden cardiac death events between ICD and medicine treatment under random effects model (Figure 4).

### 3.6 RR of cardiovascular mortality of the ICD versus medicine treatment for NICM

The *I* ^2^ was 39%, which revealed moderate heterogeneity. The test for overall effect was *Z* = 1.18 (*p* = .24) and the meta‐analysis results showed no significant difference of RR of cardiovascular mortality events between ICD and medicine treatment under the random effects model (Figure S4).

### 3.7 Log HR of cardiovascular mortality of the ICD versus medicine treatment for NICM

The *I* ^2^ was 0%, which revealed extremely low heterogeneity. The test for overall effect was *Z* = 0.49 (*p* = .62) and the meta‐analysis results showed no significant difference of log HR of cardiovascular mortality events between ICD and medicine treatment under random effects model (Figure S5).

## 4 DISCUSSION

In this updated meta‐analysis, our results also replicated the positive results of reducing the all‐cause mortality for the ICD treatment on NICM patients when compared with medicine treatment. The significant RR and log HR results of all‐cause mortality all indicated that the ICD treatment might play the role in the primary prevention of all‐cause mortality. In addition, the ICD treatment might significantly reduce the RR and log HR of sudden cardiac death. However, the lower number of enrolled studies and patients might limit the interpretations of the positive results in the primary prevention of sudden cardiac death. At last, the ICD treatment seemed not to reduce cardiovascular mortality when compared with medicine treatment on NICM patients. The low number of enrolled studies and patients suggested that further studies should focus more on cardiovascular mortality and sudden cardiac death.

Our meta‐analysis is different from the previously published meta‐analyses in several perspectives. First, our meta‐analysis enrolled the updated randomized trials, which can help the results close to the updated information. Second, our results reported significant findings of all‐cause mortality and sudden cardiac death in the presentations of RR and log HR. Third, the sample size of our meta‐analysis was larger than those of previous meta‐analytic studies.

In the current meta‐analysis, the ICD with optimal medicine treatment showed a lower risk of all‐cause mortality and sudden cardiac death. However, as we can see, most enrolled studies have a relatively higher percentage of concurrent use of angiotensin‐related medications (angiotensin‐converting enzyme inhibitors and angiotensin receptor blocker) in heart failure. In previous studies, the angiotensin‐neprilysin inhibitors showed superior clinical efficacy to reduce sudden cardiac death by 20% and improve the overall survival and reduce death due to worsening of heart failure.^17^, ^18^ However, a recent study showed that angiotensin‐neprilysin inhibitor just can improve the left ventricle ejection fraction. The overall survival was still the same as ischemic cardiomyopathy.^19^ Therefore, the possible bias from the concurrent medicine of angiotensin‐related medications might need further studies to elucidate the possible influences from the concurrent treatment of angiotensin medicine.

A recent large registered non‐randomized trial also supported the results of DANISH trial. It also replicated the findings of older NICM patients (>68 years old) would have a higher risk of overall mortality after implantation of ICD when compared to the younger NICM patients (<68 years old).^20^ It proved the limited beneficial effect of ICD on the elderly NICM patients in the “real world” clinical practice. Therefore, our meta‐analysis results of the benefits of ICD on NICM patients should be interpreted with caution on the elderly patient subgroup.

In a recent study of best practice alert suggested that patients with left ventricle ejection fraction lower than 35% should be the candidate of ICD treatment and the HR of mortality is 0.85 even with a trend (not significant) *p* value.^21^ In the most enrolled studies of the current meta‐analysis, the left ventricle ejection fraction was lower than 35%. Therefore, the benefits of ICD to reduce mortality can be consider to a strengthening reason to persuade NICM patients with left ventricle ejection fraction lower than 35% to receive the ICD treatment. In addition, the subsequent analysis showed that the improvement of left ventricle ejection fraction might be associated with improved survival.^22^ Therefore, ICD might be associated with the improvement of left ventricle ejection fraction, which might be related to the beneficial findings in all‐cause mortality and sudden cardiac death. The underlying possible mechanism for the ICD to reduce mortality might also be associated with periodic repolarization dynamics (a repolarization instability marker) in NICM patients.^23^ This kind of biomarker for the ICD benefits on NICM patients might need to be investigated in the future.

The current meta‐analysis has several limitations. First, most enrolled studies were relatively far from the current time and the variety of duration of follow‐up might influence the meta‐analysis results. However, the funnel plot showed the risk of publication bias might be low. Second, the medicine treatment might be variable in the dose and kinds of pharmacological treatment, which might influence the interpretations of our results. However, the medicine therapy for NICM with heart failure might not changed so significantly in recent years. Therefore, the medicine treatment in the enrolled studies might still represent the pattern of clinical practice. Third, the risk of device‐related infections or other adverse events might not be revealed in the current meta‐analysis. However, the lower risk of all‐cause mortality with ICD therapy might represent the clinical meaning with possibly less risk of infection mortality. Fourth, the lack of patient‐level data might also influence the interpretations of our results due to the lack a full evaluation of patient‐level covariates in across comparisons. It is impossible to confirm a possible subgroup effect related to patient age and explore the impact of enrolled studies' NHYA class and baseline mortality risk on the benefit of ICD. Fifth, the influence of cardiac resynchronization therapy in some enrolled studies should not be ignored when we tried to interpret the meta‐analysis results. Sixth, the relatively low number of enrolled studies in the meta‐analysis of sudden cardiac death and cardiovascular mortality might limit the interpretations of our findings.

## 5 CONCLUSION

In the current meta‐analysis, the ICD treatment might show a lower RR and HR of all‐cause mortality and sudden cardiac death when compared with medicine treatment. However, no significant differences were observed in cardiovascular mortality between ICD and medicine treatment. Future randomized studies of clear patient‐level data, elderly patient groups, and possible biomarkers might be warranted.

## CONFLICTS OF INTEREST

The authors declare no conflicts of interest.

## Supporting information

Supplementary information.

## Data Availability

Data available on request from the authors (The data that support the findings of this study are available from the corresponding author upon reasonable request.).

## References

[1] Bardy GH , Lee KL , Mark DB , et al. Amiodarone or an implantable cardioverter‐defibrillator for congestive heart failure. N Engl J Med. 2005;352(3):225‐237. 15659722 10.1056/NEJMoa043399

[2] Køber L , Thune JJ , Nielsen JC , et al. Defibrillator implantation in patients with nonischemic systolic heart failure. N Engl J Med. 2016;375(13):1221‐1230. 27571011 10.1056/NEJMoa1608029

[3] Al‐Khatib SM , Fonarow GC , Joglar JA , et al. Primary prevention implantable cardioverter defibrillators in patients with nonischemic cardiomyopathy: a meta‐analysis. JAMA Cardiol. 2017;2(6):685‐688. 28355432 10.1001/jamacardio.2017.0630 PMC5815025

[4] Anantha Narayanan M , Vakil K , Reddy YN , et al. Efficacy of implantable cardioverter‐defibrillator therapy in patients with nonischemic cardiomyopathy: a systematic review and meta‐analysis of randomized controlled trials. JACC Clin Electrophysiol. 2017;3(9):962‐970. 29759721 10.1016/j.jacep.2017.02.006

[5] Barakat AF , Saad M , Elgendy AY , et al. Primary prevention implantable cardioverter defibrillator in patients with non‐ischaemic cardiomyopathy: a meta‐analysis of randomised controlled trials. BMJ Open. 2017;7(6):e016352. 10.1136/bmjopen-2017-016352 PMC5726098 28637742

[6] Golwala H , Bajaj NS , Arora G , Arora P . Implantable cardioverter‐defibrillator for nonischemic cardiomyopathy: an updated meta‐analysis. Circulation. 2017;135(2):201‐203. 27993908 10.1161/CIRCULATIONAHA.116.026056 PMC5416382

[7] Luni FK , Singh H , Khan AR , et al. Mortality effect of ICD in primary prevention of nonischemic cardiomyopathy: a meta‐analysis of randomized controlled trials. J Cardiovasc Electrophysiol. 2017;28(5):538‐543. 28370885 10.1111/jce.13192

[8] Masri A , Hammadah M , Adelstein E , Jain S , Saba S . Implantable cardioverter defibrillator in non‐ischemic cardiomyopathy: a meta‐analysis of randomized controlled trials. Cardiovasc Diagn Ther. 2017;7(4):397‐404. 28890876 10.21037/cdt.2017.06.06 PMC5582055

[9] Stavrakis S , Asad Z , Reynolds D . Implantable cardioverter defibrillators for primary prevention of mortality in patients with nonischemic cardiomyopathy: a meta‐analysis of randomized controlled trials. J Cardiovasc Electrophysiol. 2017;28(6):659‐665. 28316104 10.1111/jce.13204

[10] Alba AC , Foroutan F , Duero Posada J , et al. Implantable cardiac defibrillator and mortality in non‐ischaemic cardiomyopathy: an updated meta‐analysis. Heart. 2018;104(3):230‐236. 28780582 10.1136/heartjnl-2017-311430

[11] Beggs SAS , Jhund PS , Jackson CE , McMurray JJV , Gardner RS . Non‐ischaemic cardiomyopathy, sudden death and implantable defibrillators: a review and meta‐analysis. Heart. 2018;104(2):144‐150. 28986406 10.1136/heartjnl-2016-310850

[12] El Moheb M , Nicolas J , Khamis AM , Iskandarani G , Akl EA , Refaat M . Implantable cardiac defibrillators for people with non‐ischaemic cardiomyopathy. The Cochrane database of systematic reviews. 2018;12:CD012738. 30537022 10.1002/14651858.CD012738.pub2 PMC6517305

[13] Bristow MR , Saxon LA , Boehmer J , et al. Cardiac‐resynchronization therapy with or without an implantable defibrillator in advanced chronic heart failure. N Engl J Med. 2004;350(21):2140‐2150. 15152059 10.1056/NEJMoa032423

[14] Khan SU , Ghimire S , Talluri S , et al. Implantable cardioverter defibrillator in nonischemic cardiomyopathy: a systematic review and meta‐analysis. J Arrhythm. 2018;34(1):4‐10. 29721108 10.1002/joa3.12017 PMC5828271

[15] Knobloch K , Yoon U , Vogt PM . Preferred reporting items for systematic reviews and meta‐analyses (PRISMA) statement and publication bias. J Cranio‐Maxillo‐Facial Surg. 2011;39(2):91‐92. 10.1016/j.jcms.2010.11.001 21145753

[16] Cumpston MS , McKenzie JE , Welch VA , Brennan SE . Strengthening systematic reviews in public health: guidance in the Cochrane handbook for systematic reviews of interventions. J Public Health . 2022. 10.1093/pubmed/fdac036 PMC9715291 35352103

[17] McMurray JJ , Packer M , Desai AS , et al. Angiotensin‐neprilysin inhibition versus enalapril in heart failure. N Engl J Med. 2014;371(11):993‐1004. 25176015 10.1056/NEJMoa1409077

[18] Desai AS , McMurray JJV , Packer M , et al. Effect of the angiotensin‐receptor‐neprilysin inhibitor LCZ696 compared with enalapril on mode of death in heart failure patients. Eur Heart J. 2015;36(30):1990‐1997. 26022006 10.1093/eurheartj/ehv186

[19] Abumayyaleh M , Pilsinger C , El‐Battrawy I , et al. Clinical outcomes in patients with ischemic versus non‐ischemic cardiomyopathy after angiotensin‐neprilysin inhibition therapy. J Clin Med. 2021;10(21):4989. 34768510 10.3390/jcm10214989 PMC8584412

[20] Frommeyer G , Andresen D , Ince H , et al. Can we rely on Danish? Real‐world data on patients with nonischemic cardiomyopathy from the German device registry. Heart Vessels. 2019;34(7):1196‐1202. 30607538 10.1007/s00380-018-01337-2

[21] Lee J , Szeto L , Pasupula DK , et al. Cluster randomized trial examining the impact of automated best practice alert on rates of implantable defibrillator therapy. Circulation. 2019;12(6):e005024. 31181957 10.1161/CIRCOUTCOMES.118.005024

[22] Eiger DS , Inoue LYT , Li Q , et al. Factors and outcomes associated with improved left ventricular systolic function in patients with cardiomyopathy. Cardiology J . 2021. 10.5603/CJ.a2020.0187 PMC9788743 33438181

[23] Boas R , Sappler N , von Stülpnagel L , et al. Periodic repolarization dynamics identifies ICD responders in nonischemic cardiomyopathy: a DANISH substudy. Circulation. 2022;145(10):754‐764. 34889650 10.1161/CIRCULATIONAHA.121.056464

