# Supplementary material for: The mortality for the implantable cardiac defibrillator in nonischemic cardiomyopathy: An updated systematic review and meta‐analysis
Source: Clin Cardiol. 2022 Sep 3;45(12):1163–70. doi: 10.1002/clc.23907 (PMC9748743; doi:10.1002/clc.23907)
Supplement: Supplementary file 1 — Supplementary information. [file CLC-45-1163-s001.docx]

**Supplementary Figure 1: The PRISMA flow diagram of current meta-analysis**

**The current meta-analysis followed the PRISMA guideline to identify the potentially relevant literature and screen the identified literature using abstract and title selection. The full text of screened literature was assessed to find the eligible studies and include the suitable ones for the final meta-analysis.**

**Supplementary Figure 2: The risk of bias assessment in current meta-analysis**

**The risk of bias was evaluated by the bias arising from the randomization process (D1), bias due to deviations from intended interventions (D2), bias due to missing outcome data (D3), bias in the measurement of the outcome (D4), and bias in the selection of the reported result (D5)**

**Supplementary Figure 3: The funnel plot of enrolled studies in current meta-analysis**

**The enrolled studies showed a symmetric distribution in the funnel plot, which suggested limited publication bias.**

**Supplementary Figure 4: The forest plot of RR for the meta-analysis results of cardiovascular mortality [ICD vs medicine treatment]**

**The RR of ICD treatment for events of cardiovascular mortality was not significantly lower than that of medicine treatment. A significant heterogeneity was noted.**

**Supplementary Figure 5: The forest plot of log HR for the meta-analysis results of cardiovascular mortality [ICD vs medicine treatment]**

**The log HR of ICD treatment for events of cardiovascular mortality was not significantly lower than that of medicine treatment. A significant heterogeneity was noted.**

**
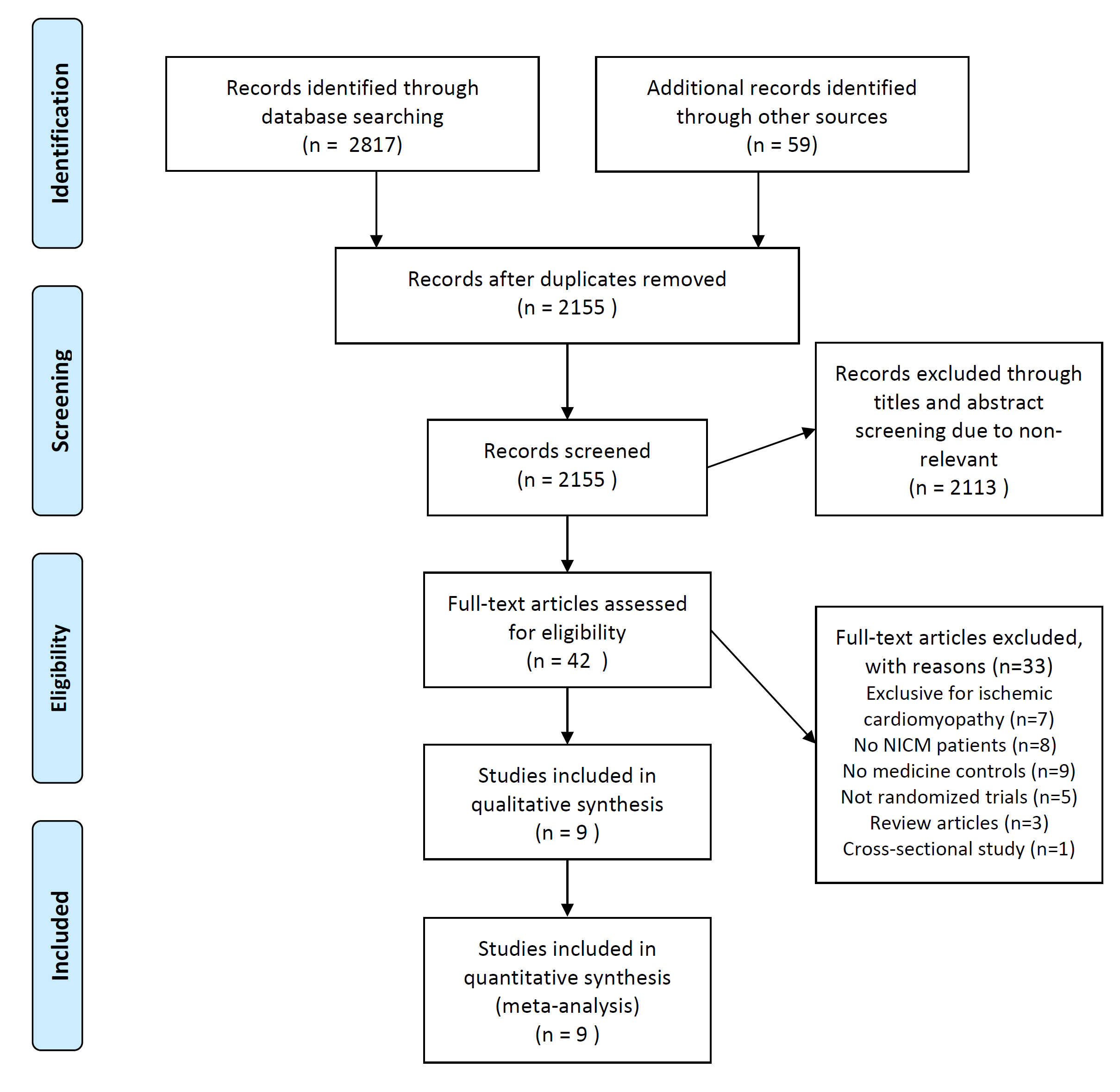
**

**Supplementary Figure 1: The PRISMA flow diagram of current meta-analysis**

**
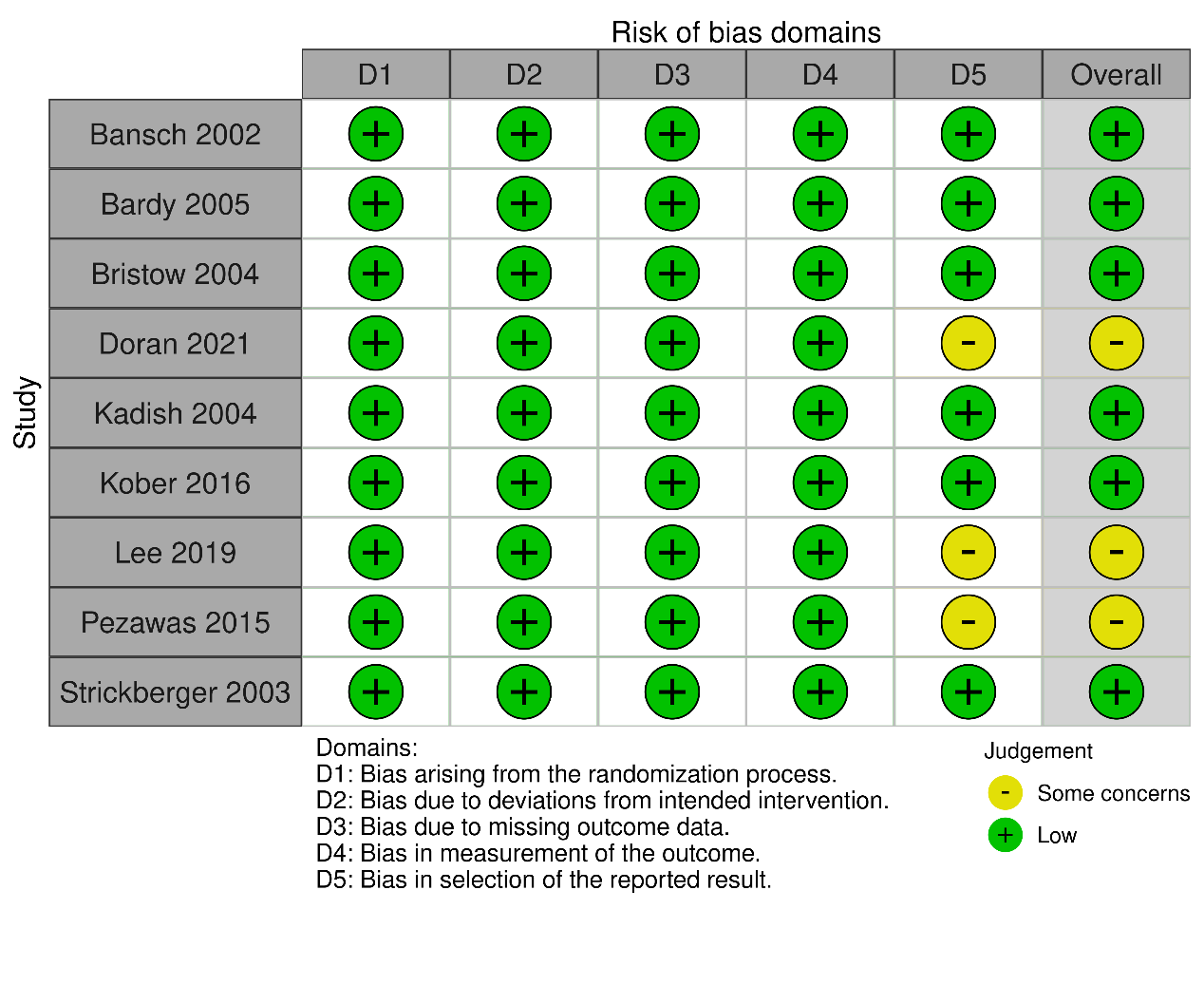
**

**Supplementary Figure 2: The risk of bias assessment in current meta-analysis**


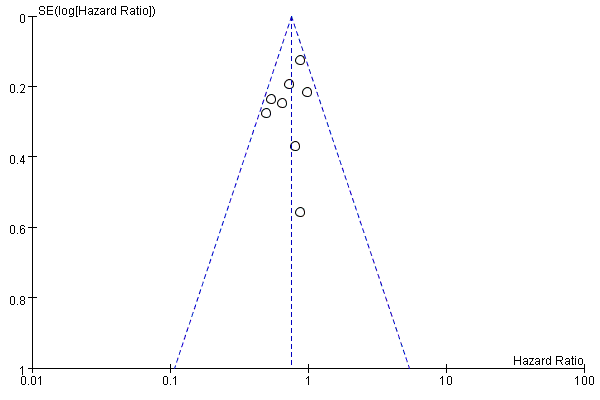


**Supplementary Figure 3: The funnel plot of enrolled studies in current meta-analysis**


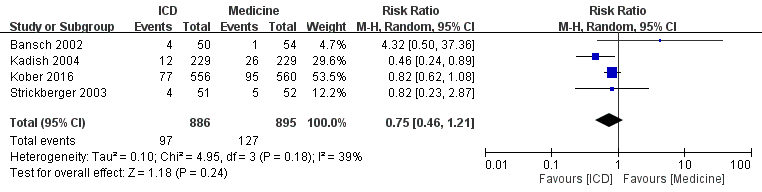


**Supplementary Figure 4: The forest plot of RR for the meta-analysis results of cardiovascular mortality [ICD vs medicine treatment]**


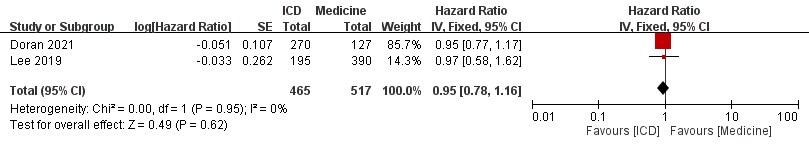


**Supplementary Figure 5: The forest plot of log HR for the meta-analysis results of cardiovascular mortality [ICD vs medicine treatment]**
